# Preservative Effects of Gelatin Active Coating Containing Eugenol and Higher CO_2_ Concentration Modified Atmosphere Packaging on Chinese Sea bass (*Lateolabrax maculatus*) during Superchilling (−0.9 °C) Storage

**DOI:** 10.3390/molecules25040871

**Published:** 2020-02-17

**Authors:** Qianqian Zhou, Peiyun Li, Shiyuan Fang, Jun Mei, Jing Xie

**Affiliations:** 1 College of Food Science and Technology, Shanghai Ocean University, Shanghai 201306, China; m170200465@st.shou.edu.cn (Q.Z.); m180310770@st.shou.edu.cn (P.L.); m170200401@st.shou.edu.cn (S.F.); 2 National Experimental Teaching Demonstration Center for Food Science and Engineering Shanghai Ocean University, Shanghai 201306, China; 3 Shanghai Engineering Research Center of Aquatic Product Processing and Preservation, Shanghai 201306, China; 4 Shanghai Professional Technology Service Platform on Cold Chain Equipment Performance and Energy Saving Evaluation, Shanghai 201306, China

**Keywords:** active coating, Chinese sea bass, superchilling, eugenol, modified atmosphere packaging

## Abstract

The purpose of this research was to explore the fresh keeping effect of modified atmosphere packaging (MAP) with different gas ratios combined with gelatin active coatings containing eugenol on Chinese sea bass stored at −0.9 °C for 36 days. The results showed that MAP3 (60% CO_2_/10% O_2_/30% N_2_), together with gelatin active coatings containing eugenol, could prevent water loss, which maintained high field NMR, MRI, and organoleptic evaluation results. This hurdle technology could also effectively delay the bacterial reproduction, protein degradation, and alkaline accumulation, so it showed the lowest K value, total volatile basic nitrogen, free amino acids, total viable count, *Pseudomonas* spp., and H_2_S-producing bacteria, which better maintain the quality of sea bass.

## 1. Introduction

Chinese sea bass (*Lateolabrax maculatus*) is an important economic aquaculture fish and is widely distributed in China. In the last several years, the annual output of sea bass culture has reached more than 100,000 tons [1]. The consumption of sea bass has also increased due to its nutritional characteristics as well as its benefits to consumer health [2]. However, fresh sea bass is high in protein and water and is prone to corruption and deterioration in the process of transportation, storage, processing, and marketing [3,4].

Superchilling is the process of lowering the temperature of a product just below its initial freezing temperature and the proportion of water frozen is approximately 5–30% preserved within the food product [5,6,7,8]. Superchilling has been used in fish processing to help significantly increase the shelf life and has been successfully applied in the preservation of Atlantic mackerel [9], hairtail [10], olive flounder [11], and seabream [12] as well as other seafood products. Aside from the temperature control, modified atmosphere packaging (MAP) could also inhibit the growth of spoilage microflora on fish [13,14]. Zhu et al. [15] reported that superchilling (−0.7 °C) with high-CO_2_ packaging (60% CO_2_/40% N_2_) could inhibit substantial inhibition of the biochemical and microbial deterioration of catfish (*Clarias gariepinus*) muscle during storage. Parlapani et al. [16] showed that 2 °C combined with 60% CO_2_/10% O_2_/30% N_2_ could slow down the increases of total volatile basic nitrogen (TVB-N) and trimethylamine nitrogen (TMA-N) and extend the shelf life by about four days. The gas component in the package delays the metabolic process of microorganisms and inhibits the growth and development of microorganisms to enable the microorganisms in a basically dormant or semi-dormant state, which achieves the purpose of extending the shelf life of packaged food [17]. He et al. [18] reported that MAP and enzyme inhibitors could reduce the protein oxidation of tilapia muscle during ice storage. Messina et al. [19] studied atmosphere packaging (80% CO_2_/20% N_2_) in combination with UV-C radiation (106.32 mJ/cm^2^), enhancing the shelf life of rainbow trout fillets by at least twice. Yew et al. [20] found that the higher concentration of CO_2_ could inhibit the formation of biogenic amines in Indian mackerel stored at 5 ± 1 °C.

Eugenol belongs to the group of phenolic components that possess strong antimicrobial and antioxidant capacity [21,22]. It kills cells by increasing the permeability of bacterial cell membranes, which leads to the leakage of substances in the cells [23]. In our previous research [24], the addition of 0.15% eugenol combined with superchilling (−0.9 °C) could maintain the moisture and freshness of sea bass and extend the shelf life. Therefore, the objective of our research was to explore the effect of different ratios of atmosphere proportion combined with a gelatin active coating enriched with a eugenol emulsion on Chinese sea bass at −0.9 °C.

## 2. Results and Discussions

### 2.1. Microbiological Results

**Figure 1:**
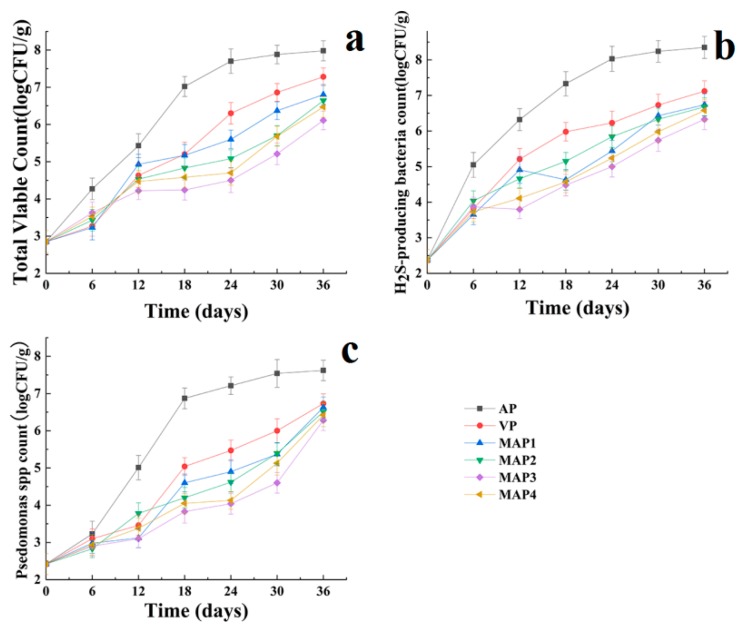
Changes in total viable count (**a**), *Pseudomonas* spp. (**b**), and H_2_S-producing bacteria (**c**) of Chinese sea bass during superchilling storage at −0.9 °C. AP: air package; VP: vacuum package; MAP1: modified atmosphere packaging with 40% CO_2_/60% N_2_; MAP2: modified atmosphere packaging with 40% CO_2_/10% O_2_/30% N_2_; MAP3: modified atmosphere packaging with 60% CO_2_/10% O_2_/30% N_2_; MAP4: modified atmosphere packaging with 60% CO_2_/40% N_2_.

Figure 1 shows the data corresponding to the growth of total viable count (TVC) of *Pseudomonas* spp. and H_2_S-producing bacteria of Chinese sea bass samples during superchilling storage at −0.9 °C for 36 days. The TVC on day 0 was 2.84 log_10_ CFU/g and increased during superchilling storage. The air package (AP) treated sea bass samples are no longer edible on the 18th day because it reaches the “shelf-life” limit of 7.0 log_10_ CFU/g for marine fish [25]. High CO_2_ and lower O_2_ concentration could inhibit the microbial growth [26] and the gelatin active coating, acting as a barrier against oxygen transfer, could inhibit the growth of aerobic bacteria [27]. The lower O_2_ could inhibit the growth of aerobic spoilage bacteria and the decomposition of trimethylamine oxide (TMAO) into trimethylamine, which can form a strong antimicrobial barrier with high CO_2_. Furthermore, eugenol possesses “moderate-strong inhibitory” characteristics to inhibit the microbial growth to extend the shelf-life of fish during storage [28,29]. Compared with AP treated sea bass samples, the gelatin active coating containing eugenol and MAP treated samples significantly inhibited the microbial growth during superchilling storage at −0.9 °C. Marchese et al. [30] reported that eugenol showed a good antimicrobial effect on Gram-negative bacteria. Similar results were also obtained in the present research toward the increase of *Pseudomonas* spp. and H_2_S-producing bacteria, which are considered as common specific spoilage organisms (SSOs) in fish during cold storage [30,31,32]. The population of these two kinds of microbial communities increased with an increase in the storage time in all samples (Figure 1b,c). The growth pattern for *Pseudomonas* spp. (Figure 1b) was similar to that of total valble count (TVC). The count of *Pseudomonas* spp. was 2.37 log_10_ CFU/g on day 0 and increased to 8.13 log_10_ CFU/g at the end of storage for the AP treated samples. However, other sea bass samples were still under 7 log_10_ CFU/g on the 36th day. The growth of *Pseudomonas* spp. was favored by packaging in air and was effectively inhibited by high CO_2_ concentrations (60%) [26]. Additionally, the lower O_2_ concentration was also probably an important factor to inhibit *Pseudomonas* growth. The initial count of H_2_S-producing bacteria in sea bass samples was 2.42 log_10_ CFU/g and also had a similar trend with that of TVC (Figure 1c). The counts of H_2_S-producing bacteria increased to 8.35, 7.12, 6.74, 6.68, 6.33, and 6.58 log_10_ CFU/g, respectively, in AP, VP, MAP1, MAP2, MAP4, and MAP3 treated samples on the 36th day. H_2_S-producing bacteria could use electron acceptors including TMAO instead of oxygen to survive under oxygen or hypoxia conditions [33] and produced proteolytic and lipolytic enzymes that led to food spoilage, which could break down proteins and produce an off-flavor to degrade sea bass quality [34]. Mohan et al. [35] reported that MAP with 60% CO_2_ could inhibit the growth of aerobic spoilage microorganisms and lower the intracellular pH value. Provincial et al. [36] studied the effects of MAP with 40, 50, and 60% CO_2_ on sea bass storage and reported that 60% CO_2_ could effectively inhibit the growth of spoilage microorganisms during cold storage. The content of O_2_ in MAP is usually kept as 5 or 10%, which could inhibit the growth of aerobic spoilage bacteria and the decomposition of TMAO into trimethylamine.

### 2.2. pH Values

**Figure 2:**
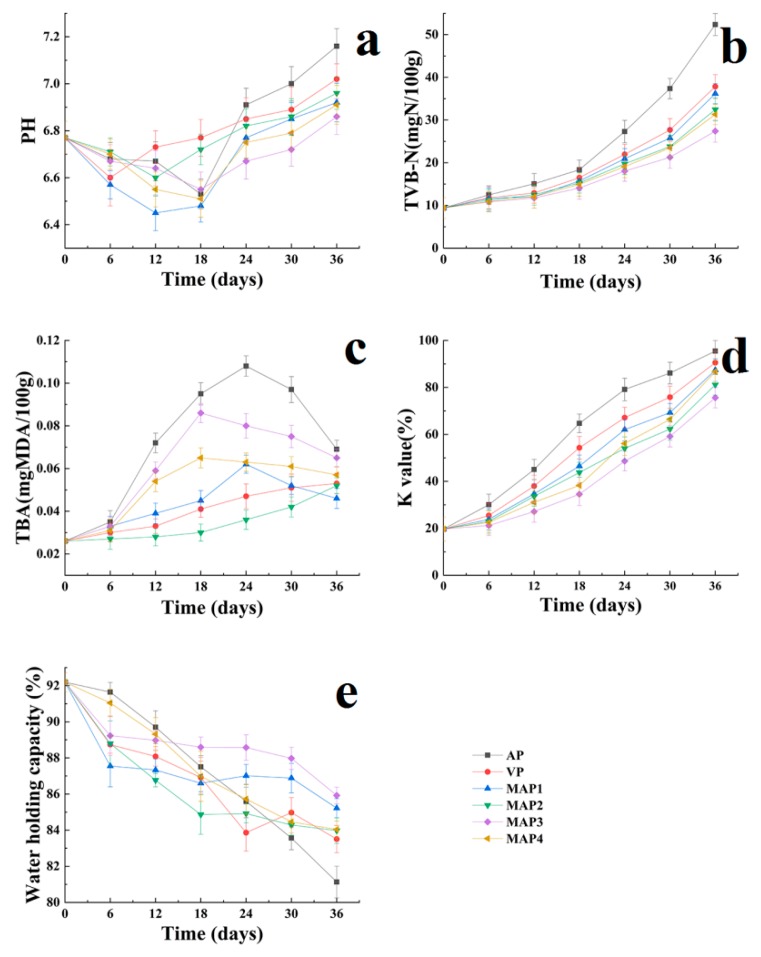
Changes in pH values (**a**), total volatile basic nitrogen (TVB-N) values (**b**), thiobarbituric acid reactive substances (TBARS) values (**c**), K values (**d**), and water holding capacity (WHC) values (**e**) of Chinese sea bass during superchilling storage at −0.9 °C. AP: air package; VP: vacuum package; MAP1: modified atmosphere packaging with 40% CO_2_/60% N_2_; MAP2: modified atmosphere packaging with 40% CO_2_/10% O_2_/30% N_2_; MAP3: modified atmosphere packaging with 60% CO_2_/10% O_2_/30% N_2_; MAP4: modified atmosphere packaging with 60% CO_2_/40% N_2_.

Microbial activity can also be measured indirectly by pH values [37]. The pH value of sea bass muscle on 0 day was 6.77 and showed a decreased tendency initially, and then increased (Figure 2a). It could be seen that the pH of AP treated sea bass samples was significantly (*p* < 0.05) higher than the other samples. The pH of the gelatin active coating containing eugenol and the MAP treated samples were at approximately 6.86–6.96, whereas the pH of the AP and VP treated samples increased drastically to pH 7.16 and 7.02 on the 36th day, respectively. The initial pH decrease was caused by the accumulation of lactic acid during glycolysis and the release of inorganic phosphates from ATP degradation [38]. However, the increase in pH values resulted from the production of volatile basic components such as biogenic amines, ammonia, and trimethylamines as a result of endogenous enzymes and bacterial propagation [39,40]. For the gelatin active coating containing eugenol and MAP treated sea bass samples, the degradation of amino acids resulting from the spoilage microorganisms was significantly inhibited and led to a decrease in the production of biogenic amines [41]. Therefore, the pH of the gelatin active coating containing eugenol and MAP treated sea bass samples was more stable than that of the AP treated samples, showing that the gelatin active coating containing eugenol and MAP treatments could help to maintain the quality of sea bass during superchilling storage.

### 2.3. Total Volatile Basic Nitrogen (TVB-N) Values

Notable TVB-N formation was also determined in Chinese sea bass samples in the present research (Figure 2b). The increase was especially obvious in AP treated sea bass samples. Higher TVB-N values in fish during storage demonstrated that nitrogenous materials accumulated from the degradation of nitrogen containing compounds including proteins and nucleic acids [42]. The TVB-N content of sea bass on 0 day was 9.4 mg in the N/100g sample, indicating the good quality of sea bass used in the present research [43]. TVB-N values increased for all samples during superchilling storage; however, this increase was faster at the later stages of storage due to storage conditions, endogenous enzymes, increased bacterial activity, and hygienic practices [44,45,46]. The AP treated sea bass samples exhibited a higher increase, reaching a value of 38.20 mg N/100g on day 30, which exceeded the maximum permissible level of 35 mg N/100 g for the spoilage initiation for fresh fish established by the European Commission [47]. The use of reduced oxygen atmosphere packaging such as VP and MAP could help in reducing the production of volatile basic components. The use of a gelatin active coating containing eugenol and MAP treatments showed significant effects on the TVB-N increase in sea bass samples during superchilling storage (*p*<0.05). At the end of storage, TVB-N values of 37.84, 36.18, 32.47, 31.32, and 27.41 mg N/100g were observed for the VP, MAP1, MAP2, MAP4, and MAP3 sea bass samples, respectively, which were still “high quality”. This protective effect of eugenol and a high CO_2_ concentration atmosphere could inhibit the growth of Gram-negative aerobic bacteria (*Pseudomonas* spp. and H_2_S-producing bacteria), which produced volatile compounds comparing to the AP treated sea bass samples [48,49].

### 2.4. Thiobarbituric Acid Reactive Substances (TBARS) Values

Thiobarbituric acid (TBA) is an indicator to evaluate the degree of lipid oxidation; TBARS were measured asmalonaldehyde (MDA) equivalent to determine the secondary oxidation products from polyunsaturated fatty acids [50]. The increase in TBA value mainly results from enzymatic hydrolysis and auto-oxidation during storage [51]. The TBARS value of sea bass samples on 0 day was 0.025 mg MDA/100 g sample (Figure 2c). Subsequently, the TBARS values of all samples showed upward trends during the early and mid-late stage, and then there were slight decreases in the AP and VP treated samples in the later stage because of the reaction of MDA with ketones and aldehydes [52]. Compared with AP treated sea bass samples, the TBARS values in the VP, MAP1, and MAP4 treated samples were relatively lower (*p* < 0.05). The concentrations of O_2_ inside these packages are about 0%, whereas that in AP is about 21%. However, the VP treated samples had no CO_2_ or O_2_, and had higher TBARS values than that of MAP4, indicating that not only the auto-oxidation of lipid, but also enzymatic hydrolysis might accelerate the increase in the TBARS value [53,54]. Meanwhile, TBARS values in MAP4 were higher than that in MAP1, which means that a higher CO_2_ concentration might have a certain promoting effect on enzymatic hydrolysis. Furthermore, on the basis of the TBARS results, the inhibiting effect of lipid oxidation may also be due to the presence of eugenol acting as an antioxidant [55].

### 2.5. Free Amino Acids (FAAs)Values

FAAs are precursors of volatile flavor compounds and are responsible for flavor development during storage [56]. In the present research, most of the FAAs showed upward trends in all Chinese sea bass samples during superchilling storage at −0.9 °C (Table 1). The major FAAs in sea bass samples were glycine, alanine, and lysine, accounting for 46.61–66.22% of total FAA content, which contribute to the desired tastes of fish [57,58]. Histidine was identified as an off-taste amino acid and accounted for 1.65–2.15% of total FAA content among all sea bass samples. On day 0, the content of histidine was 24.59 mg/100 g in the AP treated samples and decreased to 14.38 mg/100 g sea bass samples on day 36, however, the corresponding contents in MAP1, MAP2, MAP3, and MAP4 were 50.55, 48.71, 30.78, and 32.12 mg/100 g on day 30. Alanine, glycine, aspartic acid, and glutamic acid are responsible for the characteristic flavor of fish [59]. Glycine contents in AP treated samples decreased from 126.58 mg/100 g on 0 day to 58.32 mg/100 g on 36th day. Glycines content in the gelatin active coating containing eugenol and MAP treated sea bass samples had similar behaviors to the AP treated samples; however, their final contents were significantly (*p* < 0.05) lower than that of the AP treated samples. Sea bass samples treated with the gelatin active coating containing eugenol and MAP treatments also had significantly lower alanine contents during superchilling storage. In addition, the final total FAA level in the MAP treated samples was higher than that of the AP and VP treatments, which might be due to the reduction of water loss and spoilage organism counts by protective coatings and MAP treatments [60].

### 2.6. K Values

A K-value of lower than 20% is considered to be “sashimi” quality, and higher than 60% as the rejection level [61,62]. The K value in fresh sea bass samples was 19.78% and increased in all samples during superchilling storage (Figure 2d), however, the gelatin active coating containing eugenol and MAP treatments could significantly (*p* < 0.05) delay the increase in the K value, which had a similar behavior to that of the TVB-N values. The AP treated sea bass samples reached 64.68% on 18th day and MAP3 and MAP4 were still under the rejection value on 30th day, which was attributed to the higher CO_2_ concentration of MAP and the addition of eugenol. ATP and its related compounds degrade continuously after fish postmortem, where the convention from IMP to HxR is a critical reaction changing the K value under the effect of 5-nucleotidase enzymatic catalysis. Zhu et al. [53] reported that higher CO_2_ concentration inside the packaging bag could suppress the spoilage microbial activities, thus slowing down the breakdown of adenosine triphosphate. However, The K value of all the sea bass samples exceeded the rejection value at the end of storage.

### 2.7. Water Holding Capacity (WHC) Values

The WHC of fish is one the most important quality parameters affecting the weight change during storage and the tenderness and juiciness of the fish muscle [63]. WHC values of all sea bass samples decreased during superchilling storage, originating from the proteolytic activity in the muscle [64,65]. At the beginning, the decreased rate in the gelatin active coating containing eugenol and MAP treated samples was higher than that of the AP treated samples. However, at the mid-late stage of storage, the decline rate was slower and dropped from the initial value of 92.19% to about 81.13% for the VP treated samples and 83.97–85.92% for the gelatin active coating containing eugenol and MAP treated samples on day 36 (Figure 2e). The decrease in the WHC values reflected a decrease in the water protein interactions in fish muscle during superchilling storage [66].

### 2.8. Water Distribution by Low Field Nuclear Magnetic Resonance (LF-NMR) Analysis

**Figure 3:**
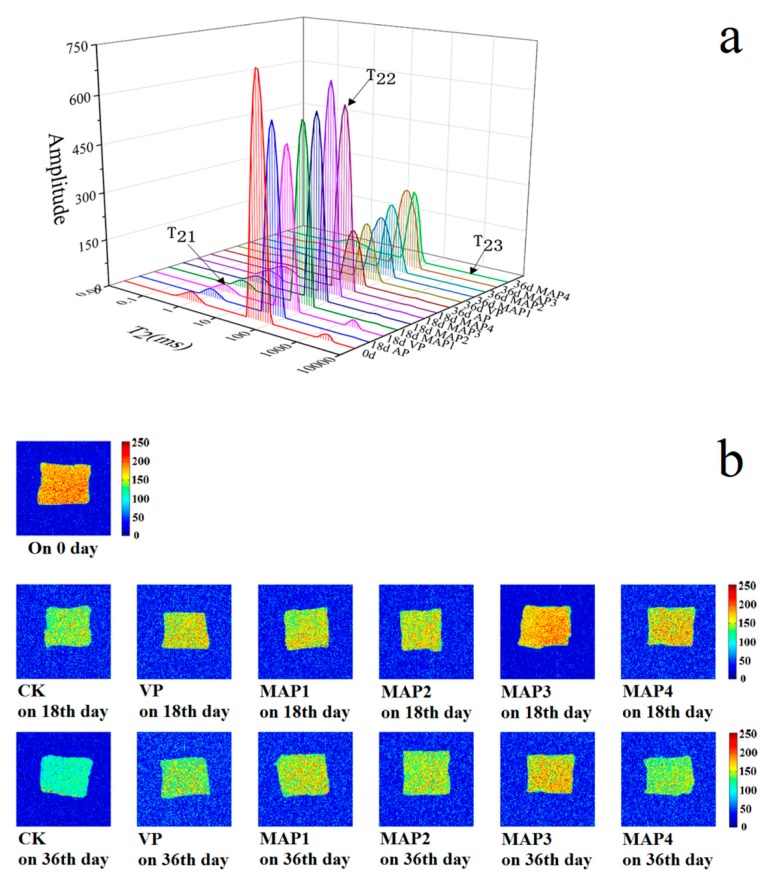
Changes in water distribution (**a**) and magnetic resonance imaging (**b**) of Chinese sea bass during superchilling storage at −0.9 °C. AP: air package; VP: vacuum package; MAP1: modified atmosphere packaging with 40% CO_2_/60% N_2_; MAP2: modified atmosphere packaging with 40% CO_2_/10% O_2_/30% N_2_; MAP3: modified atmosphere packaging with 60% CO_2_/10% O_2_/30% N_2_; MAP4: modified atmosphere packaging with 60% CO_2_/40% N_2_.

LF-NMR is an efficient technology to evaluate the freshness of fish, and magnetic resonance imaging (MRI) is also an assistive method for assessing water migration [67,68]. There is a regular reaction signal between hydrogen protons and sample water protons in low field NMR, which is closely related to the chemical reactions between proteins, lipids, and other cellular components [69]. The pT_21_, pT_22_, and pT_23_ correspond to the areas of relaxation time T_21_, T_22_, and T_23_. In this research, T_21_ showed that the bound water varied ranging from 0.79% to 1.01% during superchilling storage (Figure 3a). There was no significant difference (*p* > 0.05) among the gelatin active coating containing eugenol and the MAP treated sea bass samples during superchilling storage at −0.9 °C, indicting T_21_ could not be affected by the treated ways as well as storage time, which was due to the water entrapped within highly organized myofibril structures [70,71]. T_22_ was considered as immobile water within the myofibril [72] and pT_22_ diminished progressively during superchilling storage. T_23_ representing free water and pT_23_ increased constantly. The changes of free water in sea bass samples were more remarkable than those of bound and immobile water during superchilling storage. The AP treated sea bass samples had significant lower immobilized water (from 88.52% on 0 day to 48.53% on 36th day) than that of the MAP treated samples. MAP3 had the largest amounts of the immobilized water on 18th and 36th day, respectively, probably due to the conclusion that a higher CO_2_ concentration package decreased the diffusion rate of the immobile water to free water. Some researchers also demonstrated that water located within highly organized myofibril structures released or translated to free water based on the destruction of myofibril [69,73,74,75]. In addition, MAP treatments could retard this water migration and the change rates of T_22_ and T_23_ of sea bass samples during superchilling storage at −0.9 °C.

MRI could provide visual information of internal morphological organization and molecular distribution in fish [76]. As shown in Figure 3b, red represents a high proton density and blue represents a low proton density in the pesudo-color images. The signal intensity in each region of the sample was proportional to the content of water molecules, that is to say, the darker areas in the image meant that there were fewer water protons. There was no significant difference in the brightness of the image of sea bass samples on 0 day and the brightness was darker and bluer during superchilling storage. The color of the AP treated sea bass samples on 18th and 36th day were bluer than that of the MAP treated samples, indicating the degradation of myofibilin the AP treated sea bass samples during superchilling storage [68], while no significant visual difference was observed among the gelatin active coating containing eugenol and the MAP treated sea bass samples. The brightness of the MAP3 samples was redder compared to other samples, which demonstrated that the gelatin active coating containing eugenol combined with MAP3 treatment (60% CO_2_/10%O_2_/30% N_2_) was more suitable for quality maintenance of sea bass samples during superchilling storage and the result was in accord with the changes of LF-NMR transverse relaxation.

### 2.9. Organoleptic Properties

**Figure 4:**
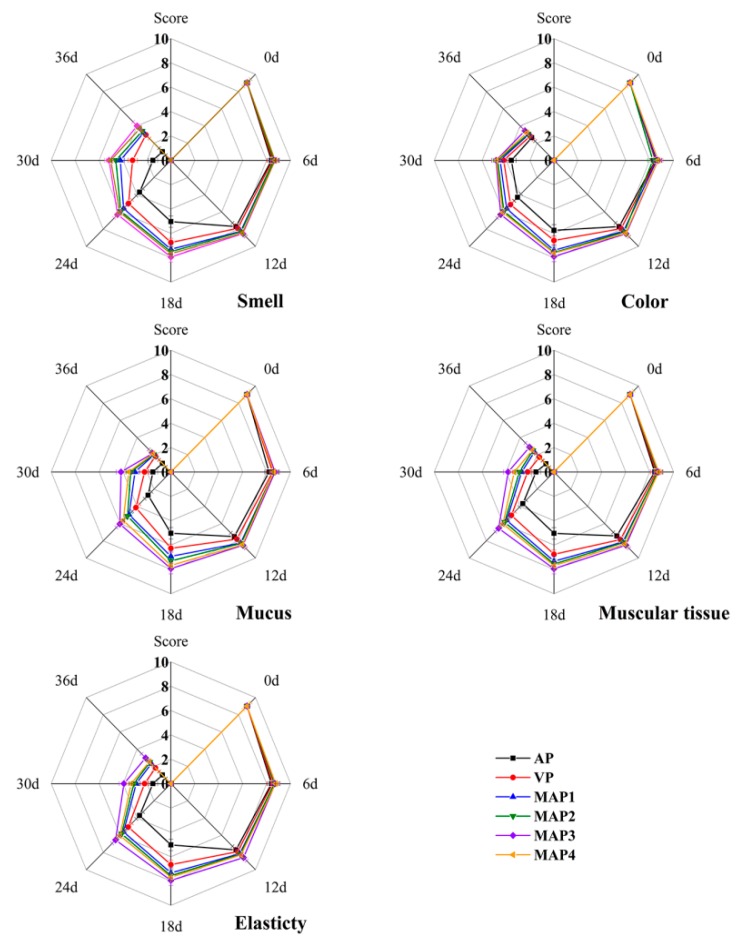
Changes in smell, color, mucus, muscular tissue, and elasticity of Chinese sea bass during superchilling storage at −0.9 °C. AP: air package; VP: vacuum package; MAP1: modified atmosphere packaging with 40% CO_2_/60% N_2_; MAP2: modified atmosphere packaging with 40% CO_2_/10% O_2_/30% N_2_; MAP3: modified atmosphere packaging with 60% CO_2_/10% O_2_/30% N_2_; MAP4: modified atmosphere packaging with 60% CO_2_/40% N_2_.

The organoleptic evaluation results including smell, color, mucus, muscular tissue, and elasticity of Chinese sea bass samples during superchilling storage at −0.9 °C for 36 days are presented in Figure 4. On 0 day, all samples had high organoleptic scores, indicating excellent quality. However, the quality of all sea bass samples decreased significantly (*p* < 0.05) with the increasing storage time. The organoleptic results showed that the gelatin active coating containing eugenol and the MAP treated sea bass samples had significantly higher scores than that of the AP and VP treated samples. On 18th day, the score of the AP treated samples was lower than 5, which was considered as an unacceptable value for sea bass samples in the present research. The gelatin active coating containing eugenol and the MAP treated sea bass samples exceeded the limitation on 30th day except for the MAP3 treated samples. Therefore, this method of treating with active coatings containing eugenol and MAP treatments could be an effective way to retard the quality deterioration and maintain the organoleptic quality of sea bass samples. Compared with the microbiological and chemical results, the organoleptic evaluation results showed some retardation in the quality evaluation of sea bass samples during superchilling storage. This was probably due to the organoleptic evaluation being subjective; and the quality deterioration appeared inside the sea bass samples first, which is usually difficult to observe in time by visual inspection [77,78]. Therefore, it should be suitable to examine the storage quality of sea bass samples by the comprehensive analysis of microbiological, chemical, and organoleptic evaluation.

## 3. Materials and Methods

### 3.1. Preparation of Gelatin Active Coatings Containing Eugenol Emulsions

In our previous research [24], the eugenol concentration used in the present research was 1.5% (*v*/*v*) and the microencapsulated emulsion was prepared according to Li et al. [79]. A total of 150 μL eugenol and 750 mg β-cyclodextrin (β-CD) were stirred mechanically in a beaker and then 5 g Tween-80 was added to make them homogeneously dispersed. Then, deionized water was added continuously to get a final volume of 100 mL and the emulsion was obtained by continuous stirring for another 6 h. The emulsion was homogenized with a rotor-stator homogenizer (HR-6, Huxi Industrial Co. Ltd., Shanghai, China) at 15,000 rpm for 5 minand the microencapsulated emulsion was obtained. Gelatin (6.0% *w*/*w*, bloom value at 240–270, BBI Life Science, Shanghai, China) and glycerol (1.5% *v*/*w*) were dissolved in prepared microencapsulated eugenol emulsions (3 L) at 50 °C and stirred for 4 h. Then, the mixture was ultrasonically treated (XEB-1000-P, Xiecheng Ultrasonic Equipment Co. Ltd., Shandong, China) at 800 W for 10 min and degassed under vacuum.

### 3.2. Preparation of Sea Bass and Sample Treatments

A total of 111 live sea bass with an average length of 40 ± 3 cm were obtained from a fish market in Nanhui new town (Shanghai, China), transported to the laboratory, and rested for two days in a tank supplied with oxygenated freshwater. After that, fish were slaughtered by using the ice slurry methods. After the fish were stunned by ice slurry methods, they were immediately gutted; the gill and viscera of the sea bass were removed and washed with sterilized 1% NaCl solutions. Three random sea bass samples were used to determine the basic quality profiles on 0 day. The remaining samples were immersed in the freshly prepared microencapsulated eugenol emulsions (ratio of solution to sea bass samples, 3:1, *v*/*w*) for 60 s, then allowed to drain at 4 °C around 45 min to form the active coatings on the sea bass surface. Then, the coated samples were divided into six batches and submitted to the following packages: (1) packaged in the presence of air (AP); (2) vacuum package (VP); (3) MAP1 (40% CO_2_/60% N_2_); (4) MAP2 (40% CO_2_/10% O_2_/30% N_2_); (5) MAP3 (60% CO_2_/10% O_2_/30% N_2_); and (6) MAP4 (60% CO_2_/40% N_2_). After that, all samples were stored at −0.9 ± 0.1 °C in a thermo tank (BPS-250CB, Yiheng Thermostatic Chamber, Shanghai, China). Sea bass samples were taken randomly for analysis on days 0, 6, 12, 24, 30, and 36, respectively.

### 3.3. Microbiological Analysis

Representative 10 g sea bass muscle was homogenized with 90 mL of stroke physiological saline solution and then subjected to serial dilutions. The total viable counts, *Pseudomonas* spp., and H_2_S-producing bacteria were cultivated on plate count agar medium, cetrimide agar medium, and iron agar medium, respectively, at 30 °C for 48 h [31].

### 3.4. pH Value Determination

For the determination of pH value, we referred to the method of Messina et al. [19]. Ten grams of sea bass muscle and 90 mL deionized water were homogenized at a speed of 5000 rpm for 5 min at 4 °C and then stood for 30 min. The supernatant was used to determine the pH value with a pH meter.

### 3.5. Water Distribution and Migration

The proton relaxation experiments were carried out according to Li et al. [73]. The dorsal part of the sea bass muscle was cut into cubes (2.5 × 2 × 1.3 cm, about 5 g) and wrapped in polyethylene films. Transverse relaxation T_2_ measurements were determined with a LF-NMR analyzer (Niumag MesoMR23-060H.I, Suzhou, China) with a proton resonance frequency of 20 MHz. MRI experiments were performed to gain pseudo-color images of the proton density weight of sea bass. The acquisition parameters were as follows: slice width was 3 mm, time repetition was 2000 times, and time echo was 15 ms.

### 3.6. WHC Determination

WHC values were determined as per the method of Zang et al. [80]. Three grams of sea bass muscle from the dorsal part was centrifuged at 3000 g for 10 min at 4 °C. The percentage of retained water after centrifugation was expressed as WHC.

### 3.7. TVB-N Values Determination

The TVB-N values were determined by the microtitration method [81] and expressed as mg N/100 g of sea bass muscle.

### 3.8. TBARS Value Determination

Lipid oxidation was monitored by the evaluation of TBARS [82] and expressed as mg of MDA/kg of sea bass muscle. Five grams of sea bass muscle was homogenized with 20 mL of 20% trichloroacetic acid solution, and rested for 1 h. After being centrifuged at 11,960 ×*g* for 10 min at 4 °C, 5 mL of the supernatant was mixed with 5 mL TBA solution (0.02 M) and heated in 100 °C for 40 min. Then, the mixture was cooled to room temperature with an ice bath and the absorbance was measured at 532 nm with a spectrophotometer (Evolution 220, Thermo Fisher Scientific, Waltham, MA, USA).

### 3.9. Determination of Adenosine Triphosphate (ATP) Related Compounds

ATP-related compounds were determined proposed by Wang et al. [83] with a reversed-phase high performance liquid chromatogram (RP-HPLC) method (Waters 2695, Milford, CT, USA). Sample preparation method was as follows: 5 g minced sea bass muscle homogenized with 10 mL10% perchloric acid (PCA) and centrifuged at 8000 g for 15 min at 4 °C. The precipitate was stirred with 10 mL 5% PCA and centrifuged at 8000 g for 10 min at 4 °C twice. The supernatant pH was adjusted to 6.5 after the supernatant was merged and added with 15 mL of distilled water. After 30 min, take the supernatant fixed in a 50 mL volumetric bottle with ultrapure water. Finally, the supernatant was filtered with a 0.22 µm membrane and applied to the RP-HPLC procedure. The chromatographic conditions were as follows: column: Shimadzu ODS–3 C18 (4.6 mm × 250 mm, 5 mm); mobile phase: A–20 mmol/L KH_2_PO_4_:20 mmol/L K_2_HPO_4_ (*v*/*v* 1:1), adjusted to pH 6.5 with phosphoric acid; B–Methanol; column temperature: 30; injection volume: 10 mL; detection wavelength: 254 nm; flow rate: 1.0 mL/min; gradient: 0–6 min 100%A, 6–15 min B increases linearly to 8%, 15–20 min B increases linearly to 35%, 20–22 min 35%B,22–24 min B decreases linearly to 0%, 24–30 min 100% A. The K value was calculated as follows:

(1) $$K\;\mathrm{value}\;(\%)=\frac{\mathrm{HxR}+\mathrm{Hx}}{\mathrm{ATP}+\mathrm{ADP}+\mathrm{AMP}+\mathrm{IMP}+\mathrm{HxR}+\mathrm{Hx}}\;\times 100\;$$

### 3.10. FAA Analysis

FAAs were monitored by the method of Liu et al. [84] with some modifications. 5g of mashed sea bass muscle and 15 mL of 15% cold trichloroacetic acid were homogenized at 10,000 rpm for 5 min and left to stand at 4 °C for 2 h. After being centrifuged at 5980 ×*g* for 15 min at 4 °C, 5 mL of the supernatant was diluted with deionized water to 10mL. Then, the mixture was filtered through a 0.22 μm filter and analyzed by an amino acid analyzer (Hitachi L-8800, Tokyo, Japan). Parameter conditions of the automatic amino acid analyzer are as follows: separation column (4.6 mm × 60 mm); resin: cation exchange resin; separation column temperature: 57 °C; detection wavelength: 570 nm (proline: 440 nm); injection 20 μL; buffer flow rate: 0.35 mL/min; reaction solution: ninhydrin reagent; flow rate 0.35 mL/min; unit temperature 135 °C.

### 3.11. Organoleptic Properties

The Quality Index Method (QIM) developed by Lanzarin et al. [85] and Freitas et al. [86] was used for organoleptic evaluation with some modifications. The odor, color, mucus, elasticity, and muscle tissue of the sea bass were scored. Each parameter had 10 simple descriptors where 10 represented the best quality and a lower score indicated poorer quality. Twelve trained participators joined in the organoleptic evaluation. Organoleptic evaluation was done on each test day. At each sampling time, the participators were asked to state whether the sea bass samples were acceptable or not to determine the shelf life.

### 3.12. Statistical Analysis

The one-way analysis of variance (ANOVA) procedure was applied for multiple comparisons by SPSS 22.0, and the results were expressed as means ± standard deviation.

## 4. Conclusions

The gelatin active coating containing eugenol combined with MAP treatments were conducted to evaluate the effects on the quality improvement of Chinese sea bass samples during superchilling storage at −0.9 °C for 36 days. The results of physicochemical and microbiological analyses indicated that the MAP3 (60% CO_2_/10% O_2_/30% N_2_) treated sea bass samples maintained better quality results during superchilling storage, which was mainly due to the high CO_2_ and lower O_2_ concentrations of the MAP conditions that could effectively inhibit the growth of spoilage microorganisms to extend the shelf life. Furthermore, the gelatin active coating containing eugenol also promotes the shelf-life and safety of sea bass for its broad spectrum of antimicrobial activity and resistance to oxidation. Therefore, gelatin active coatings containing eugenol combined with 60% CO_2_/10% O_2_/30% N_2_ MAP treatments are suitable for maintaining the quality of Chinese sea bass samples during superchilling storage where an extended storage period may be required.

## Figures and Tables

**Table 1:** Changes in the free amino acid content of Chinese sea bass during superchilling storage at −0.9 °C.

Time	Samples	Free Amino Acids					
		Aspartic Acid	Threonine	Serine	Glutamic Acid	Glycine	Alanine
On day 0		3.71 ± 0.36	12.36 ± 0.75	25.32 ± 0.88	11.79 ± 0.43	126.58 ± 0.99	81.80 ± 0.62
On 18th day	AP	4.33 ± 0.26b	29.45 ± 0.96ab	27.4 ± 0.82b	17.86 ± 0.53e	72.45 ± 0.67e	87.62 ± 1.77c
	VP	1.88 ± 0.22d	26.04 ± 0.81bc	24.28 ± 0.76c	19.82 ± 0.65d	78.58 ± 0.78d	90.14 ± 0.83c
	MAP1	3.20 ± 0.41c	33.10 ± 0.87a	28.10 ± 0.73b	23.34 ± 0.59c	82.24 ± 0.64c	94.01 ± 1.85b
	MAP2	3.46 ± 0.19c	30.71 ± 0.83a	30.14 ± 0.81a	23.94 ± 0.38c	88.32 ± 0.98b	95.54 ± 0.83b
	MAP3	6.54 ± 0.38a	30.87 ± 0.58a	22.12 ± 0.72d	27.34 ± 0.43a	99.30 ± 0.49a	103.14 ± 0.88a
	MAP4	4.37 ± 0.25b	22.47 ± 0.61c	25.49 ± 0.73c	25.65 ± 0.65b	89.64 ± 1.26b	97.75 ± 2.93b
On 36th day	AP	3.81 ± 0.14d	16.89 ± 0.43d	11.47 ± 2.2e	20.87 ± 2.7c	58.32 ± 1.07e	59.10 ± 3.43f
	VP	4.07 ± 0.55d	20.06 ± 0.58c	18.40 ± 1.7d	27.70 ± 4.8ac	71.77 ± 3.75d	70.81 ± 1.52e
	MAP1	5.04 ± 0.35d	33.90 ± 0.55a	70.29 ± 5.3a	38.06 ± 4.5b	97.23 ± 2.6c	87.85 ± 2.08d
	MAP2	10.23 ± 0.68b	33.67 ± 0.84a	54.81 ± 6.2b	41.90 ± 6.33b	101.08 ± 3.09c	100.81 ± 1.72c
	MAP3	13.49 ± 0.62a	28.48 ± 0.61b	39.60 ± 7.3c	55.83 ± 5.32a	153.56 ± 0.62a	117.46 ± 1.89a
	MAP4	8.96 ± 0.58c	33.36 ± 0.71a	43.27 ± 5.2c	51.89 ± 6.84a	134.75 ± 1.72b	106.86 ± 2.17b
		Valine	Methionine	Isoleucine	Leucine	Tyrosine	Phenylalanine
On day 0		7.66 ± 0.25	4.48 ± 0.37	5.52 ± 0.28	8.76 ± 0.29	4.30 ± 0.21	4.68 ± 0.28
On 18th day	AP	11.59 ± 0.52b	6.51 ± 0.50b	7.20 ± 0.24bc	11.61 ± 0.32b	7.65 ± 0.18d	7.25 ± 0.23d
	VP	9.51 ± 0.46e	4.83 ± 0.36c	6.20 ± 0.75d	9.74 ± 0.43d	5.44 ± 0.36a	5.68 ± 0.46e
	MAP1	13.28 ± 0.19a	6.71 ± 0.16b	8.91 ± 0.29a	10.59 ± 0.28c	9.56 ± 0.17b	9.60 ± 0.26b
	MAP2	10.34 ± 0.34d	7.17 ± 0.63ab	7.12 ± 0.88bc	10.95 ± 0.25c	7.87 ± 0.23b	7.09 ± 0.31d
	MAP3	11.05 ± 0.27bc	7.69 ± 0.82a	7.25 ± 0.27bc	12.73 ± 0.31a	8.47 ± 0.25c	9.67 ± 0.27b
	MAP4	10.56 ± 0.22cd	6.58 ± 0.27b	8.17 ± 0.18ab	12.36 ± 0.28a	10.22 ± 0.31a	9.88 ± 0.35a
On 36th day	AP	11.14 ± 0.27d	6.42 ± 0.32d	6.70 ± 1.57c	10.1 ± 0.34d	5.01 ± 0.29b	7.03 ± 0.48d
	VP	10.26 ± 0.33e	6.33 ± 0.48d	7.84 ± 1..32c	10.01 ± 0.65d	9.75 ± 0.36a	5.96 ± 0.44e
	MAP1	20.44 ± 0.23b	11.85 ± 0.37c	17.21 ± 1.59a	28.54 ± 0.44a	19.98 ± 0.54b	15.28 ± 0.48a
	MAP2	19.87 ± 0.29	12.13 ± 0.31bc	13.84 ± 1.39b	23.45 ± 0.63b	16.58 ± 0.65b	12.81 ± 0.39b
	MAP3	23.08 ± 0.18a	15.32 ± 0.35a	12.84 ± 0.96b	21.63 ± 0.48c	13.59 ± 0.53b	11.18 ± 0.57c
	MAP4	18.22 ± 0.28c	12.74 ± 0.31b	17.56 ± 1.84a	28.84 ± 0.61a	18.54 ± 0.62	15.55 ± 0.48a
		Lysine	Histidine	Arginine	Proline	Total	
On day 0		78.26 ± 0.44	24.59 ± 0.45	14.20 ± 0.32	8.09 ± 0.26	432.90 ± 3.37	
On 18th day	AP	36.49 ± 2.758c	29.84 ± 0.77a	15.24 ± 0.35c	7.61 ± 0.58b	410.82 ± 4.76d	
	VP	31.68 ± 0.47d	25.63 ± 0.32b	12.14 ± 0.43e	5.82 ± 0.83c	380.99 ± 5.84e	
	MAP1	45.94 ± 0.98b	23.84 ± 0.58c	17.19 ± 0.48a	10.23 ± 0.24a	450.25 ± 3.28b	
	MAP2	36.23 ± 0.37c	22.96 ± 0.38c	13.13 ± 0.35d	9.55 ± 0.85a	434.04 ± 4.98c	
	MAP3	47.96 ± 2.01a	17.23 ± 0.53d	16.28 ± 0.23b	8.02 ± 0.67b	462.27 ± 4.22a	
	MAP4	28.04 ± 0.29e	18.33 ± 0.66c	11.64 ± 0.42e	5.42 ± 0.33c	415.27 ± 5.25d	
On 36th day	AP	34.21 ± 0.74e	14.38 ± 0.63e	8.25 ± 1.35 d	5.60 ± 0.64d	326.17 ± 4.18e	
	VP	29.51 ± 0.87f	14.66 ± 0.59e	9.87 ± 0.94d	6.28 ± 0.68d	344.17 ± 5.34d	
	MAP1	81.22 ± 0.56c	50.55 ± 0.46a	33.46 ± 0.69a	23.88 ± 0.72a	648.66 ± 4.52c	
	MAP2	103.46 ± 1.04a	48.71 ± 0.63b	27.48 ± 0.73b	21.36 ± 0.83b	655.09 ± 5.83bc	
	MAP3	91.46 ± 0.73b	30.78 ± 0.58d	23.87 ± 0.53c	18.43 ± 0.77c	682.33 ± 5.07a	
	MAP4	75.19 ± 0.87d	32.12 ± 0.52c	28.52 ± 0.81b	22.01 ± 0.68b	660.26 ± 4.78b	

Different lower case letters in different groups from same day indicate significant differences (*p* < 0.05). AP: air package; VP: vacuum package; MAP1: modified atmosphere packaging with 40% CO_2_/60% N_2_; MAP2: modified atmosphere packaging with 40% CO_2_/10% O_2_/30% N_2_; MAP3: modified atmosphere packaging with 60% CO_2_/10% O_2_/30% N_2_; MAP4: modified atmosphere packaging with 60% CO_2_/40% N_2_.
